# Identification and validation of an E2F-related gene signature for predicting recurrence-free survival in human prostate cancer

**DOI:** 10.1186/s12935-022-02791-9

**Published:** 2022-12-05

**Authors:** Cheng Yang, Lei Chen, Qingsong Niu, Qintao Ge, Jiong Zhang, Junyue Tao, Jun Zhou, Chaozhao Liang

**Affiliations:** 1 Department of Urology, The First Affiliated Hospital of Anhui Medical University, Hefei, China; 2 Institute of Urology, Anhui Medical University, Hefei, China; 3 Anhui Province Key Laboratory of Genitourinary Diseases, Anhui Medical University, Jixi Road 218, Shushan District, Hefei City, 230022 Anhui Province People’s Republic of China

**Keywords:** Prostate cancer, Biochemical recurrence, E2F, Gene signature, TCGA, GEO

## Abstract

**Background:**

It is well-established that biochemical recurrence is detrimental to prostate cancer (PCa). In the present study, we explored the mechanisms underlying PCa progression.

**Methods:**

Five cohorts from The Cancer Genome Atlas (TCGA) and Gene Expression Omnibus databases were used to perform gene set variation analysis (GSVA) between nonrecurrent and recurrent PCa patients. We obtained the intersection of pathway enrichment results and extracted the corresponding gene list. LASSO Cox regression analysis was used to identify recurrence-free survival (RFS)-related significant genes and establish an RFS prediction gene signature and nomogram. MTT and colony formation assays were conducted to validate our findings.

**Results:**

The E2F signaling pathway was activated in recurrent PCa patients compared to nonrecurrent patients. We established an E2F-related gene signature for RFS prediction based on the four identified E2F-related genes (CDKN2C, CDKN3, RACGAP1, and RRM2) using LASSO Cox regression in the Memorial Sloan Kettering Cancer Center (MSKCC) cohort. The risk score of each patient in MSKCC was calculated based on the expression levels of CDKN2C, CDKN3, RACGAP1, and RRM2. PCa patients with low-risk scores exhibited higher RFS than those with high-risk scores. Receiver operating characteristic (ROC) curve analysis validated the good performance and prognostic accuracy of the E2F-related gene signature, which was validated in the TCGA-prostate adenocarcinoma (TCGA-PRAD) cohort. Compared to patients with low Gleason scores and early T stages, PCa patients with high Gleason scores and advanced T stages had high-risk scores. Moreover, the E2F-related gene signature-based nomogram yielded good performance in RFS prediction. Functional experiments further confirmed these results.

**Conclusions:**

The E2F signaling pathway is associated with biochemical recurrence in PCa. Our established E2F-related gene signature and nomogram yielded good accuracy in predicting the biochemical recurrence in PCa.

**Supplementary Information:**

The online version contains supplementary material available at 10.1186/s12935-022-02791-9.

## Introduction

Prostate cancer (PCa) represents a common urinary malignancy, with nearly 1.3 million new cases and 359,000 cancer-specific deaths worldwide in 2018, ranking as the 2nd most common and 5th leading cause of cancer-related death in men [1]. Although the past decade has witnessed a significant progression in therapeutic strategies for PCa, including radical prostatectomy (RP), radiation therapy (RT), and androgen deprivation therapy (ADT), the prognosis for PCa patients with biochemical recurrence (BCR) remains unsatisfactory [2, 3]. BCR refers to elevated serum prostate-specific antigen (PSA) levels after RP, leading to increased cancer-specific mortality [4]. Therefore, improving the early prediction rates of biochemical recurrence is essential to enhance patient outcomes.

For PCa patients who undergo RP and RT, the 10-year BCR rate is approximately 20–50% [5]. An increasing body of evidence suggests that biochemical recurrence significantly reduces the median overall survival of PCa patients following RP [3, 6]. In this respect, PCa patients with BCR tend to develop distant metastases without specific clinical symptoms, with no consensus on the optimal management strategy for this patient population, accounting for the poor survival rates [5]. Hence, understanding the mechanisms underlying biochemical recurrence in PCa could provide new insights into diagnosis and treatment.

It is widely acknowledged that the E2F family, consisting of 8 subclasses, including E2F1–E2F8, is the major transcriptional regulator of cell cycle progression and cell proliferation [7–9]. Recent evidence has shown that E2F1–E2F3 act as transcriptional activators, and E2F4–E2F8 as transcriptional repressors [7]. Overwhelming evidence suggests that E2Fs regulate the cell cycle and participate in many other physiological processes, such as cell apoptosis, DNA damage repair, and autophagy, which play crucial roles in tumor progression [10, 11]. Abnormal expression of E2Fs has been associated with a poorer prognosis in malignancies, including lung and ovarian cancers [12, 13]. In PCa, activation of the E2F signaling pathway is involved in the DEP domain-containing 1-mediated tumor growth and bone metastasis [14]. Thus, we speculated that E2F-related genes might be associated with the BCR of PCa and could be harnessed as biomarkers.

In recent years, bioinformatics analysis has been widely used to identify gene signatures or tumor markers to improve the diagnosis, treatment, and prognosis of this patient population [15]. In this study, we found that the E2F signaling pathway was associated with the BCR of PCa. Moreover, an E2F-related gene signature was established to predict BCR in PCa patients, which revealed that patients with high-risk scores had shorter RFS. Functional experiments further validated our findings in bioinformatics analysis. Altogether, our findings substantiated the important role of the E2F signaling pathway in the BCR, which may provide potential diagnostic and prognostic value for PCa.

## Materials and methods

### Patients and sample collection

The Cancer Genome Atlas (TCGA) prostate adenocarcinoma (TCGA-PRAD) data were downloaded from UCSC Xena. Other datasets, including Memorial Sloan Kettering Cancer Center (MSKCC, GSE21032), GSE116918, GSE70768, and GSE70769, were obtained from the Gene Expression Omnibus (GEO) database. The gene expression profiles of the five datasets were preprocessed as previously described [16]. PCa tissues and adjacent noncancerous tissues were collected from PCa patients for immunohistochemistry analysis after prostatectomy at the First Affiliated Hospital of Anhui Medical University. Our study was approved by the Ethics Committee of the First Affiliated Hospital of Anhui Medical University, and written informed consent was obtained from the participants (Approval No. PJ 2021-12-24).

### Gene set variation analysis (GSVA)

Gene set variation analysis (GSVA), a gene set enrichment method, is used to assess variations in pathway activities in a certain population [17]. Fifty hallmark gene sets were obtained from the Gene Set Enrichment Analysis (GSEA) database. We performed GSVA between the recurrence and nonrecurrence groups using the “GSVA” package in R software (Version 3.4.3) [17, 18]. Commonly activated or suppressed signaling pathways were selected and overlapped to identify significant and stable pathway gene sets.

### Establishment of the prognostic E2F-related gene signature with LASSO Cox regression using the MSKCC cohort

Based on the results achieved above, we obtained the gene list of the “HALLMARK_E2F_TARGETS” pathway from the GSEA database (https://www.gsea-msigdb.org/gsea/msigdb/cards/HALLMARK_E2F_TARGETS.html), with 420 founder gene sets included. Univariate Cox regression was performed to screen RFS-related gene candidates in the MSKCC cohort. Subsequently, we used LASSO Cox regression analysis to establish an optimal RFS prediction signature for PCa patients based on these candidates using the “glmnet” package in R [19]. Briefly, LASSO regression was used to identify the E2F-related genes associated with the biochemical recurrence of PCa, and Cox regression was performed to obtain the corresponding coefficients of each gene. The risk formula was calculated based on the expression levels of the candidate genes and their corresponding coefficients, which was expressed as follows: = ${\sum }_{i =1}^{n} ({\mathrm{coef}}_{i}\times {\mathrm{Expr}}_{i})$, where ${\mathrm{Expr}}_{i}$ is the expression level of the candidate gene in patient $i$, and ${\mathrm{coef}}_{i}$ is the coefficient of gene $i$. The risk score of the E2F-related gene signature of each patient was obtained based on the risk formula.

### Evaluation of the E2F-related gene signature for RFS prediction in PCa patients using the MSKCC cohort

Based on the risk score of the E2F-related gene signature, an equal number of PCa patients from the MSKCC cohort were allocated into low- and high-risk groups. We used Kaplan–Meier (K–M) survival analysis to assess survival differences between PCa patients from different risk groups. A heatmap was generated to visualize the expression of the four E2F-related genes in the low- and high-risk groups using the “pheatmap” package in R software. The “survival” package in R was used to perform a two-sided log-rank test. Moreover, time-dependent receiver operating characteristic (ROC) curve analysis was adopted to obtain the area under the curve (AUC) for 1-year, 3-year, 5-year, and 10-year RFS and to estimate the performance of the E2F-related gene signature in RFS prediction using the “survivalROC” package in R [20].

### Validation of the prognostic value of the E2F-related gene signature in the TCGA-PRAD cohort

To determine the clinical application value of the established gene signature derived from the MSKCC cohort, the TCGA-PRAD cohort was utilized to validate the gene signature. Kaplan–Meier analysis, log-rank test, and time-dependent ROC curves were performed to demonstrate the significance and accuracy of RFS prediction using the TCGA-PRAD cohort.

### Association of the prognostic signature with other clinicopathological characteristics

The association between the risk score and pathological T stage and Gleason score was analyzed using the *t*-test. In the MSKCC and TCGA datasets, K-M survival analysis was performed for PCa patients with pathological stages T3 + T4 and Gleason score ≥ 7.

### Establishment and validation of the RFS-predicting nomogram based on the E2F-related gene signature

To explore the advantages of our E2F-related gene signature, ROC curve analysis was performed between our E2F gene signature and four previously published PCa-related gene signatures [21–24]. Among these, Yang et al. constructed a gene signature with 28 hypoxia-related genes to predict BCR in localized PCa [21]; Liu et al. built a cancer stem cell-related gene signature comprising 13 genes to predict early BCR in PCa [22]; Cuzick et al. established a cell cycle progression (CCP) score that integrated 31 CCP genes to predict BCR in PCa [23]. Finally, Zhang et al. established a prostate cancer stemness model (PCS) with 13 genes to predict progression-free survival in PCa [24]. Multivariate Cox regression was performed to identify RFS-related clinicopathological characteristics in the MSKCC cohort, which was used to establish the E2F-related gene signature-based RFS-predicting nomogram using the “Regplot” package in R for application of the E2F-related gene signature in clinical practice. The calibration curve and decision curve analysis (DCA) were used to assess the performance of the RFS-predicting nomogram. Based on our nomogram, each patient in the TCGA cohort obtained a nomogram score, and a Kaplan–Meier analysis was conducted between PCa patients with low and high scores. DCA and time-dependent ROC analysis were used to evaluate the predictive ability of the nomogram scores.

### Cell culture, transfection, and antibodies

Human PCa cell lines (PC3 and C4-2) were purchased from the American Type Culture Collection (ATCC, Manassas, VA, USA) and cultured in Dulbecco’s modified Eagle’s medium (DMEM, Gibco, USA) with 10% fetal bovine serum (FBS, Cat# 10270-106, Gibco, UK) and penicillin–streptomycin combination solution (10 kU/mL penicillin and 10 mg/mL streptomycin, Cat# PB180120, Procell, Wuhan, China) at 37 °C and 5% CO_2_. PCa cells were transfected with small interfering RNAs (siRNAs) (General biological system, Anhui, China) using Lipofectamine 3000 reagent (Lot# 2307436, Invitrogen, USA). The sequences of si-cyclin-dependent kinase inhibitor 2C (si-CDKN2C), si-Rac GTPase-activating protein 1 (si-RACGAP1), and the nontargeting control (NC) are shown in Additional file 1: Table S1. The antibodies used were as follows: anti-CDKN2C (Cat# ab192239, Abcam, Cambridge, UK), anti-RACGAP1 (Cat# ab134972, Abcam, Cambridge, UK), anti-β‐actin (Cat# AF7018; Affinity, OH, USA), and anti-β‐tubulin (Cat# AF7011, Affinity, OH, USA). The secondary antibodies included goat anti‐rabbit for CDKN2C, RACGAP1, and β-actin (Cat# AS014, ABclonal, Wuhan, China) and goat anti-mouse for β-tubulin (Cat# S0002, Affinity, OH, USA).

### Immunohistochemistry (IHC) analysis

IHC was performed as described in our previous study [25]. Briefly, after fixing the tissues with 4% formalin for 48 h overnight at 4 °C, the PCa and adjacent noncancerous tissues were embedded in paraffin and then sliced into 5-μm thick sections. Subsequently, the tissue sections were dewaxed and dehydrated with xylene and 100%, 95%, and 75% alcohol solutions. Then, antigen retrieval and endogenous peroxidase blocking with 0.3% hydrogen peroxide were conducted, and sections were incubated with primary antibodies against CDKN2C and RACGAP1 (1:100) overnight. The tissue sections were washed with phosphate-buffered saline (PBS, pH 7.2) and incubated with secondary antibodies (1:400, Cat# PV-6000, Zsbio, Beijing, China) at room temperature. After incubating the sections with horseradish peroxidase and 3,3′-diaminobenzidine (DAB, Cat# ZLI-9018, Zsbio, Beijing, China), the sections were counterstained with hematoxylin. Images were obtained under a light microscope (Product model# CX43, Olympus, Japan).

### Western blotting

Western blotting was performed as described in our previous study [26]. Total protein was extracted from cells using RIPA lysis buffer (Sigma, USA), and the concentrations were determined using a BCA assay kit (Cat# P0012S, Beyotime, Shanghai, China). After mixing with loading buffer and boiling for 10 min, samples were loaded onto sodium dodecyl sulfate (SDS) polyacrylamide gels, and then the proteins were transferred onto NC membranes. The membranes were blocked in 5% nonfat milk (Yili Industrial Group, Inner Mongolia, China) for 1 h at room temperature. After incubation with specific primary antibodies overnight at 4 °C, the membranes were incubated with the corresponding secondary antibodies (1:5000) for 1.5 h at room temperature. Immune complexes were visualized using an ECL reagent (Cat# P0018AS, Beyotime, Shanghai, China). The optical density values were quantified using ImageJ software (NIH, Bethesda, USA).

#### MTT

PC3 cells (3000 cells per well) or C4-2 cells (5000 cells per well) were seeded into 24-well plates and cultured for 24 h. Cell growth was determined at 0, 24, 48, and 72 h after transfection. Briefly, 50 μL of MTT (3-(4,5-dimethylthiazol-2-yl)-2,5-diphenyltetrazolium-bromide, 5 mg/mL, Cat# 3580GR001, BioFroxx, Germany) was added to each well and incubated at 37 °C for 1.5 h. After removing the medium, the formazan crystals were dissolved in 1 mL of DMSO for 10 min. Relative cell growth was determined based on the optical density value of each well.

### Colony formation

Cells were seeded into 6-cm dishes at a density of 1000 cells per well, and colony formation was assessed on day eight. The cell culture medium was changed regularly, and methanol was used to fix the colonies for 15 min. The colonies were then washed with PBS and stained with 1% crystal violet. The number of colonies was quantified using ImageJ software (NIH, Bethesda, USA). We counted the colony numbers by a deep-learning based counting tool, CFU.Ai (https://www.cfu.ai/), it help us to obtain the exact number for each well.

## Results

### The E2F signaling pathway is significantly activated in the progression from nonrecurrence to recurrence of PCa

**Fig. 1:**
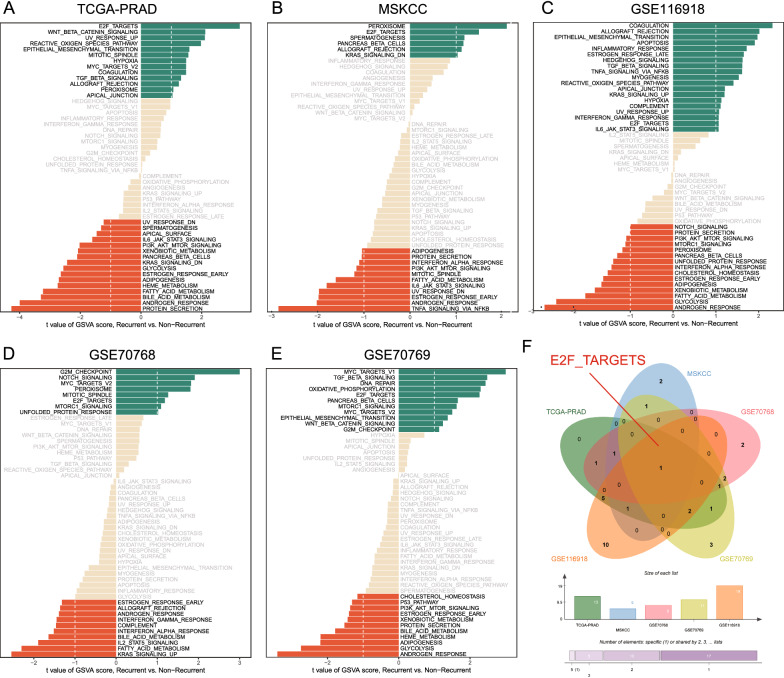
Results of GSVA between recurrent and nonrecurrent PCa patients from five datasets. The activated or suppressed pathways in the TCGA-PRAD (**A**), MSKCC (**B**), GSE116918 (**C**), GSE70768 (**D**), and GSE70769 (**E**) cohorts were identified using GSVA, and the Venn diagram (**F**) showed that E2F_TARGETS was the commonly identified pathway among the GSVA results of the five PCa cohorts

GSVA of hallmark gene sets between recurrent and nonrecurrent PCa patients was performed in the TCGA-PRAD, MSKCC, GSE116918, GSE70768, and GSE70769 cohorts (Fig. 1A–E). The results showed that the E2F signaling pathway was significantly activated in the progression from nonrecurrence to recurrence (Fig. 1F), and the GSVA results of each dataset are displayed in Additional files 2–6: Tables S2–S6. The results of GSVA analyses of the five databases (TCGA-PRAD, MSKCC, GSE116918, GSE70768, and GSE70769) showed that E2F-related genes might be valuable prognostic candidates for PCa patients.

### Establishment of an E2F-related gene-based RFS prediction signature in the MSKCC cohort

**Fig. 2:**
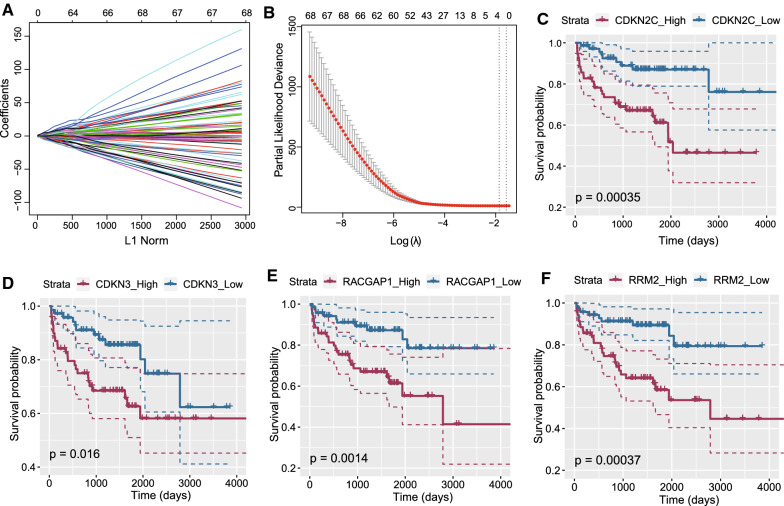
Establishment of an E2F-related RFS predictive gene signature using LASSO Cox regression in the MSKCC cohort. LASSO Cox regression identified four E2F-related genes, CDKN2C, CDKN3, RACGAP1, and RRM2, and the E2F-related gene signature was established based on these four genes (**A, B**). Based on the median expression levels of CDKN2C, CDKN3, RACGAP1, and RRM2, PCa patients were divided into high and low groups, and PCa patients with high expression levels of these four genes exhibited poorer recurrence-free survival time validated by log-rank survival test (**C–F**)

**Fig. 3:**
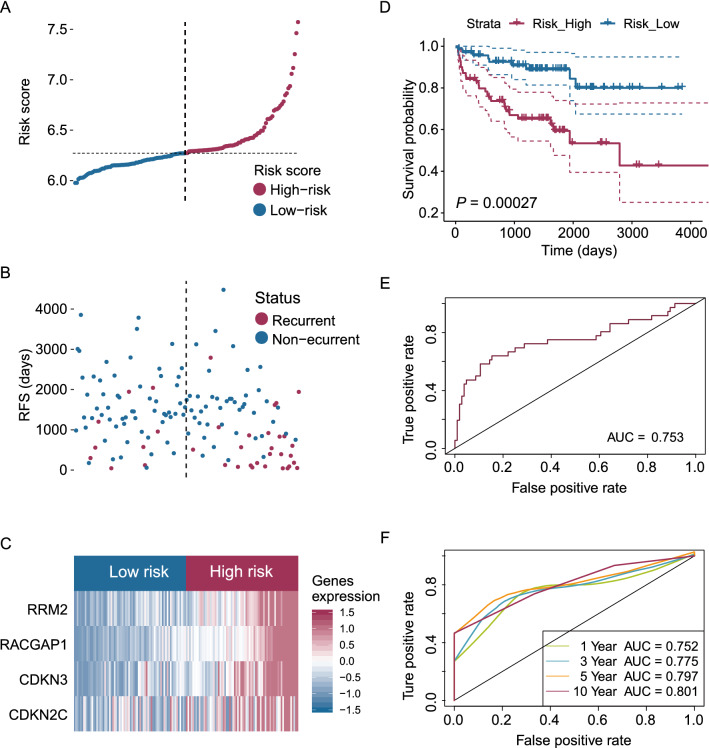
Prediction of RFS using the E2F-related gene signature of PCa patients in the MSKCC cohort. The distribution of risk scores (**A**) and disease statuses (**B**) in PCa patients, and the dashed line represents the median value of the risk score. The heatmap showed that the four identified genes, CDKN2C, CDKN3, RACGAP1, and RRM2, were highly expressed in PCa patients with high-risk scores compared to patients with low-risk scores (**C**), and patients with higher risk scores exhibited poorer RFS (**D**). The ROC curve analysis indicated the good performance of the E2F-related gene signature in RFS prediction (**E**), and the 1-, 3-, 5-, and 10-year time-dependent ROC curves indicated the good predictive value of the E2F-related gene signature (**F**)

We performed a univariate Cox regression analysis to identify RFS-related gene candidates based on the MSKCC cohort. As shown in Additional file 7: Tables S7, 74 genes were significantly associated with RFS in PCa patients (*P* < 0.01). Subsequently, LASSO and Cox regression analyses identified four E2F-related genes, CDKN2C, RACGAP1, cyclin-dependent kinase inhibitor 3 (CDKN3), and ribonucleotide reductase small subunit M2 (RRM2) (Fig. 2A, B) that were negatively associated with RFS in PCa patients (Fig. 2C–F). Based on LASSO and Cox regression analysis, we established the following risk formula: CDKN2C expression * 0.127464855559568 + CDKN3 expression * 0.178478657444053 + RACGAP1 expression * 0.581698523389747 + RRM2 expression * 0.115712688666379. The median risk score obtained from the gene signature divided these patients into low-risk and high-risk groups (Fig. 3A, B). A heatmap showed the expression differences of the four E2F-related genes between high- and low-risk PCa patients (Fig. 3C). The survival analysis results indicated that PCa patients with a low-risk score exhibited increased survival compared to patients with a high-risk score (Fig. 3D). ROC curve analysis demonstrated the good performance of the established model, with an AUC of 0.753 (Fig. 3E). Time-dependent ROC curve analysis revealed that the prognostic accuracy of the E2F-related gene signature was 0.752, 0.775, 0.797, and 0.801 at 1, 3, 5, and 10 years, respectively (Fig. 3F). These results indicated the potential clinical application value of our E2F-related gene-based RFS prediction signature.

### Validation of the E2F-related gene signature in the TCGA-PRAD cohort

**Fig. 4:**
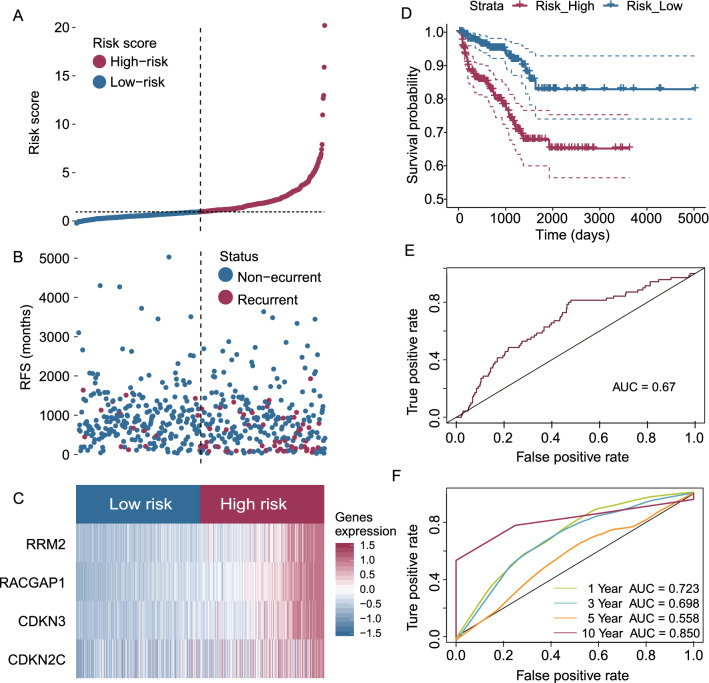
Prediction of RFS using the E2F-related gene signature of PCa patients in the TCGA-PRAD cohort. The distribution of risk scores (**A**) and disease statuses (**B**) in PCa patients, and the dashed line represents the median value of the risk score. The heatmap showed that the four identified genes were highly expressed in PCa patients with high-risk scores compared to patients with low-risk scores (**C**), and patients with higher-risk scores exhibited poorer RFS (**D**). The ROC curve analysis showed the good performance of the E2F-related gene signature in predicting the RFS of PCa patients (**E**), and the 1-, 3-, 5-, and 10-year time-dependent ROC curves indicated the accuracy of the E2F-related gene signature in RFS prediction (**F**)

The TCGA-PRAD cohort was used to estimate the RFS-predicting gene signature via the formula generated from the MSKCC cohort. Similarly, the median risk score obtained from the established gene signature was regarded as the cut-off value in the TCGA-PRAD cohort (Fig. 4A, B). A heatmap was used to visualize the expression levels of the four E2F-related genes between high- and low-risk patients in the TCGA-PRAD cohort (Fig. 4C). K–M analysis showed that PCa patients with high-risk scores exhibited poorer survival outcomes than those with low-risk scores (Fig. 4D). The ROC curve analysis displayed adequate goodness of fit of the established E2F-related gene signature, with an AUC of 0.670 (Fig. 4E). The predictive value of the E2F-related gene signature was confirmed by time-dependent ROC curve analysis, with AUC values of 0.723, 0.698, 0.558, and 0.85 for 1-, 3-, 5-, and 10-year survival, respectively (Fig. 4F).

### Subgroup analysis of the prognostic value of the E2F-related gene signature

**Fig. 5:**
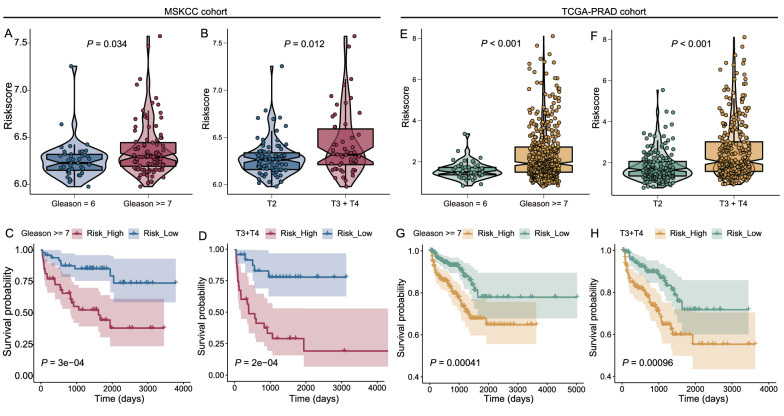
Subgroup analysis of risk scores in PCa patients with different Gleason scores and T stages. PCa patients with Gleason score ≥7 and T3 + T4 stage had higher risk scores than patients with Gleason score = 6 and T2 stage in the MSKCC cohort (**A, B**) and TCGA-PRAD (**E, F**) cohort. PCa patients with higher risk scores exhibited poorer RFS than patients with lower risk scores in PCa patients with a Gleason score ≥7 and T3 + T4 stage in the MSKCC cohort (**C, D**) and TCGA-PRAD (**G, H**) cohort

In the MSKCC cohort, PCa patients with higher Gleason scores (≥7) and advanced T stages (T3 + T4) exhibited higher risk scores than those with lower Gleason scores and early T stages (Fig. 5A, B). We also revealed that in PCa patients with Gleason scores ≥7 and T3 + T4 stages, the E2F-related signature showed satisfactory prognostic value for RFS (Fig. 5C, D). We observed similar results in the TCGA-PRAD cohort. Patients with Gleason scores ≥7 and T3 + T4 stages had higher risk scores than patients with Gleason scores <7 or T2 stages (Fig. 5E, F), and the RFS prognostic value of the E2F-related signature was good in advanced PCa in the TCGA-PRAD cohort (Fig. 5G, H).

### The performance of the E2F-related gene signature-based nomogram in predicting RFS

**Fig. 6:**
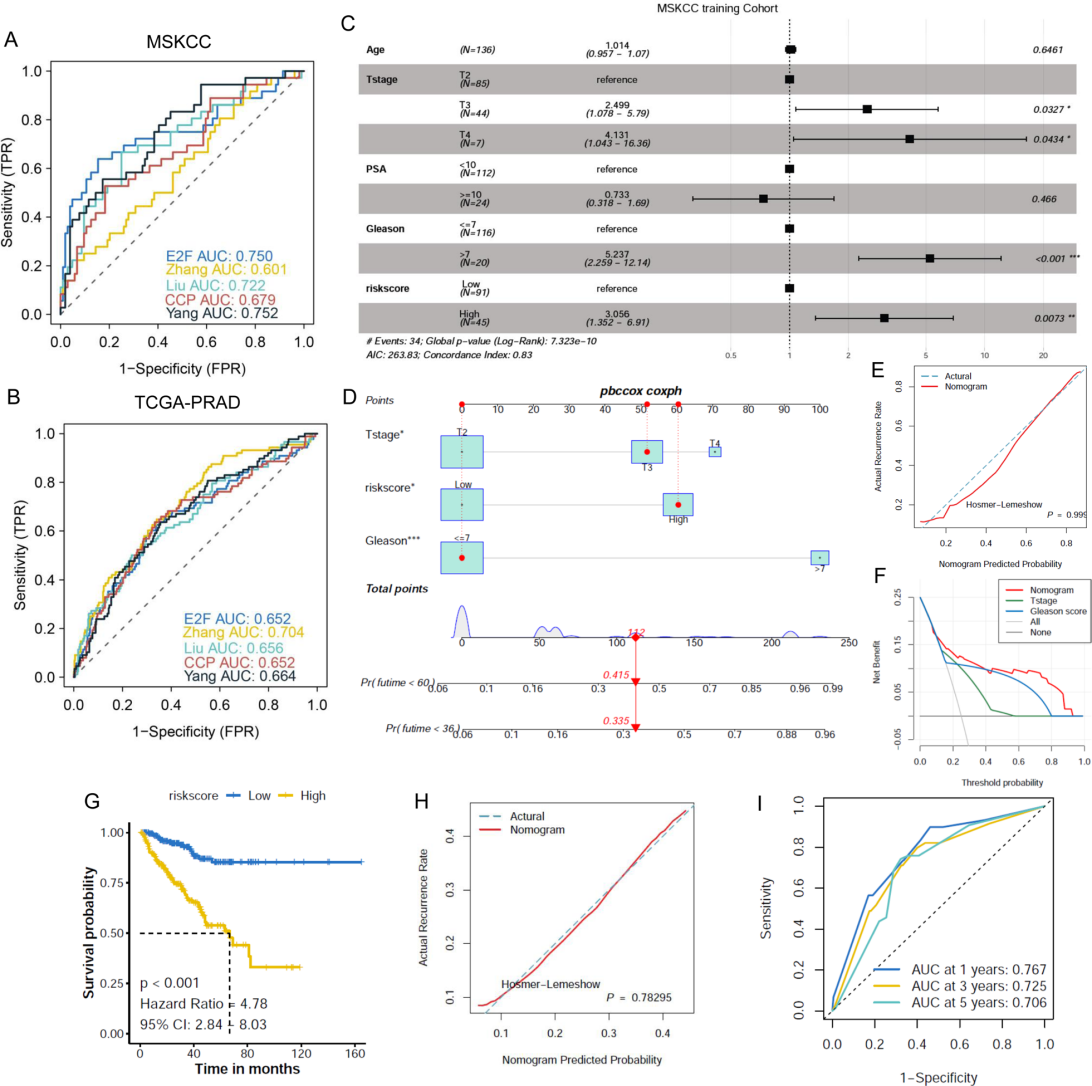
Establishment and validation of the E2F-related gene signature-based nomogram for RFS prediction. The ROC curve analysis showed the good performance of the E2F-related gene signature in predicting the RFS of PCa patients compared with the other four documented gene signatures in the MSKCC and TCGA-PRAD cohorts (**A, B**). Multivariate Cox regression indicated that T stage, Gleason score, and risk score of the E2F-related gene signature were associated with the RFS of PCa patients (**C**), and the RFS-predicting nomogram that integrated T stage, Gleason score, and risk score was established to predict the RFS of PCa patients (**D**). The calibration curve showed good agreement of the nomogram in RFS prediction compared to the actual RFS of PCa patients, and the DCA analysis visualized the net benefit of the E2F-related gene signature-based nomogram compared to T stage or Gleason score alone (**E, F**). The K–M plot showed that PCa patients with high points derived from the RFS-predicting nomogram exhibited poorer RFS than patients with low points (**G**), and the calibration curve displayed good agreement of the point in RFS prediction compared to the actual RFS of PCa patients. The time-dependent ROC curve demonstrated that the points exhibited good predictive value of the point in RFS prediction of PCa patients (**H, I**)

ROC analysis showed that the AUCs of the E2F-related gene signature were 0.750 and 0.652 in the MSKCC and TCGA-PRAD cohorts, respectively. Compared to four other previously published PCa-related gene signatures that integrated more than ten genes [21–24], our E2F-related gene signature yielded good performance predicting RFS, with only four genes included (Fig. 6A, B). The results of multivariate Cox regression analysis indicated that the T stage, Gleason score, and risk score of the E2F-related gene signature were associated with the RFS of PCa patients in the MSKCC cohort (Fig. 6C). An RFS-predicting nomogram that integrated the T stage, Gleason score, and risk score was established, and the calibration curve showed good agreement between the predicted and actual 3/5-year RFS rates. DCA emphasized the advantages of our E2F-related gene signature-based nomogram in RFS prediction compared to the T stage and Gleason score (Fig. 6D–F). Each patient obtained a score derived from the RFS-predicting nomogram in the TCGA-PRAD cohort, and patients with high scores exhibited poor prognoses (Fig. 6G). The calibration curve and time-dependent ROC curve analyses demonstrated the good performance of the nomogram scores, with AUCs of 0.767, 0.725, and 0.706 for 1/3/5-year survival, respectively (Fig. 6H–I).

### Functional validation

**Fig. 7:**
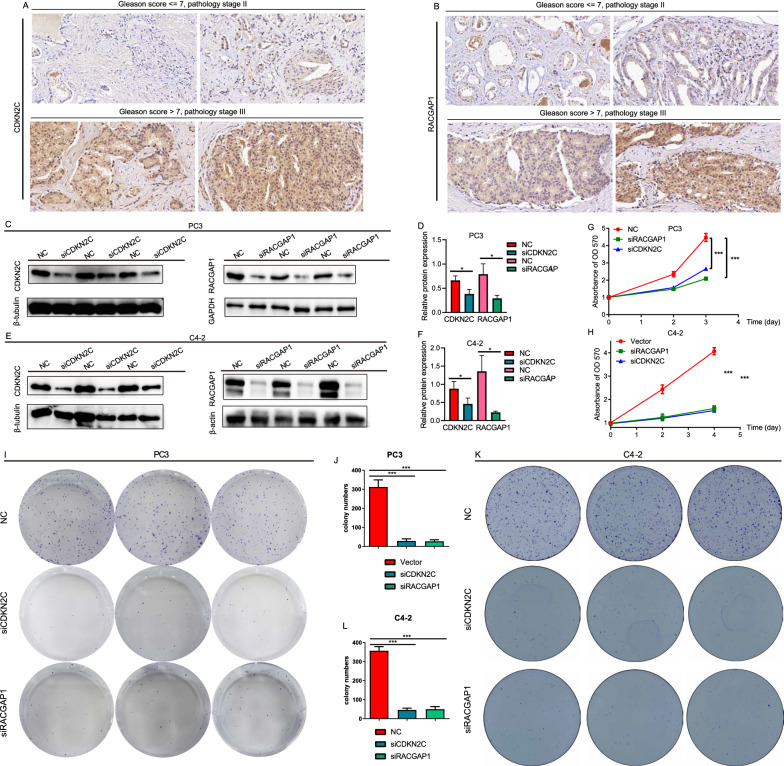
Functional validation of CDKN2C and RACGAP1 in PCa cells. Immunohistochemistry analysis showed that CDKN2C and RACGAP1 were more significantly upregulated in PCa patients with Gleason score >7 and pathology stage III compared to patients with Gleason score ≤7 and pathology stage II (**A, B**). Western blotting and bar graph show the expression and densitometric of CDKN2C and RACGAP1 knockdown in PC3 cells (**C, D**) and C4-2 cells (**E–F**). MTT (**G–H**) and colony formation assays (**I–L**) revealed that the proliferation and colony formation abilities of PC3 and C4-2 cells were significantly suppressed in response to the knockdown of CDKN2C and RACGAP1

Based on the importance of E2F-related genes in PCa, we retrieved references aiming to determine the functional evidence of CDKN2C, CDKN3, RACGAP1, and RRM2 in PCa. It has been shown that CDKN3 and RRM2 are highly expressed in PCa and promote the growth of PCa cells, while depletion of CDKN3 and RRM2 exhibits the reverse effects [27, 28]. Nonetheless, few studies have focused on the function of CDKN2C and RACGAP1 in PCa. In this study, the IHC assay showed that CDKN2C and RACGAP1 expression was increased in PCa tissues compared with adjacent tissues (Additional file 8: Fig. S1A, B). Additionally, CDKN2C and RACGAP1 were highly upregulated in PCa patients with Gleason score >7 and pathological stage III than in patients with Gleason score ≤7 and pathological stage II (Fig. 7A, B). Then, we transfected siCDKN2C and siRACGAP1 into PC3 and C4-2 cells to suppress the expression of CDKN2C and RACGAP1. Western blotting showed that the expression of CDKN2C and RACGAP1 was downregulated by knockdown of CDKN2C and RACGAP1 (Fig. 7C–F). The results of MTT demonstrated that the growth of PC3 and C4-2 cells was significantly suppressed in response to the suppression of CDKN2C and RACGAP1 (Fig. 7G, H). Similarly, we found that the colony-forming abilities of PC3 and C4-2 cells were inhibited by si-CDKN2C and si-RACGAP1 (Fig. 7I–L). Taken together, these results suggested that these critical gene markers in the E2F signaling pathway are prognostic indicators of PCa.

## Discussion

In the current study, we explored the relationship between the E2F signaling pathway and the BCR in PCa.Importantly, we found that the E2F signaling pathway was activated in biochemically recurrent PCa patients. Four E2F-related genes (CDKN2C, CDKN3, RACGAP1, and RRM2) were associated with BCR in PCa.The E2F-related gene signature was established based on these four genes and exhibited good performance in RFS prediction. The risk score was associated with the Gleason score and T stage of PCa, and patients with higher risk scores have a poor prognosis. Our nomogram based on the E2F-related gene signature yielded good performance in predicting the RFS of PCa patients. Moreover, knockdown of CDKN2C and RACGAP1 suppressed the proliferation and colony formation of PCa cells, indicating their oncogenic roles in PCa. Taken together, we demonstrated that the E2F signaling pathway was associated with BCR in PCa, and our established E2F-related gene signature could predict RFS, suggesting it has huge prospects for application in the management of biochemically recurrent PCa patients.

The occurrence of BCR is associated with the metastasis and cancer-specific mortality of PCa, accounting for the poor prognosis of patients; hence, it is essential to develop optimal management approaches for biochemically recurrent PCa patients [5]. Clinically, PCa patients with similar clinical and pathological parameters often experience different clinical outcomes despite receiving similar treatment, indicating that a heterogeneous genetic background is associated with PCa progression [29]. Although high-throughput transcriptome profiling techniques have been widely used to identify biomarkers for improving the diagnosis of PCa, patient prognosis and risk stratification are mainly dependent on clinicopathological parameters, including PSA, pathological T stage, and the Gleason score [30]. Therefore, identifying effective biomarkers for the early diagnosis and prognostic prediction of PCa is essential.

The transcriptional activities of the E2F family play a significant role in regulating the cell cycle and apoptosis, and several members of the E2F family possess dual functions, including tumor-suppressive activities and oncogenic properties [31]. An increasing body of evidence suggests that abnormal expression of E2F members is associated with a poorer prognosis in lung and ovarian cancers [12, 13]. In castration-resistant prostate cancer (CRPC), E2F1 was found to interact physically with androgen receptors and the transcription cofactor HES6, which enhanced E2F1 activity and promoted cell proliferation [32]. Knockdown of E2F1 suppressed the proliferation, invasion, and migration of PC3 cells [33]. Additionally, E2F5 expression levels positively correlated with the clinical stage and Gleason score of PCa, and patients with high E2F5 levels were more likely to suffer from metastasis and PSA failure [34]. Similarly, GSVA between PCa patients with and without BCR in five PCa cohorts (GSE70768, GSE70769, GSE116918, TCGA-PRAD, and MSKCC) showed that the E2F signaling pathway was significantly activated in PCa patients with BCR, highlighting the importance of the E2F pathway. To explore the role of the E2F pathway in biochemically recurrent PCa, we used LASSO regression to identify RFS-related genes, and four genes, CDKN2C, CDKN3, RACGAP1, and RRM2, were identified, which were negatively associated with RFS in PCa patients in the MSKCC cohort. Cox regression was performed to construct the E2F-related gene signature based on the four identified genes to predict biochemical recurrence in PCa, which was validated in the TCGA-PRAD cohort. The risk score of each PCa patient was calculated based on the E2F-related gene signature. Our results revealed that PCa patients with high-risk scores exhibited poor RFS compared to patients with low-risk scores, and similar results were found during subgroup analysis. The ROC and time-dependent ROC curve analyses confirmed the good performance and adequate accuracy of the E2F-related gene signature in RFS prediction of PCa patients, which was further validated in the TCGA-PRAD cohort. Subsequently, we found that PCa patients with high Gleason scores and advanced T stages exhibited higher risk scores. To explore the clinical usefulness of the E2F-related gene signature, we established an RFS-predicting nomogram that integrated the T stage, Gleason score, and risk score of the E2F-related gene signature in the MSKCC cohort. The calibration curve and DCA demonstrated the good performance of the E2F-related gene signature-based nomogram in RFS prediction of PCa patients, which was validated in the TCGA-PRAD cohort. Hence, the E2F signaling pathway may play a vital role in the progression and prognosis of PCa.

CDKN3 plays a key role in regulating the cell cycle and tumor progression. Current evidence suggests that CDKN3 is positively associated with TNM stages but negatively with disease-specific survival of nasopharyngeal carcinoma [35]. In PCa, highly expressed CDKN3 was associated with a poorer patient prognosis, and depletion of CDKN3 inhibited the growth and invasion of PC3 cells. As expected, overexpression of CDKN3 enhanced proliferation, which was also confirmed in nude mice [27]. It is well-established that RRM2, a subunit of ribonucleotide reductase (RNR), plays an important role in DNA synthesis and repair. Overexpression of RRM2 in various tumors is associated with poor outcomes by regulating the cell cycle [36, 37]. Mazzu et al. found that RRM2 exerted oncogenic effects on PCa, and RRM2 levels were positively associated with Gleason scores and metastasis in PCa, associated with poor prognosis in these patients [28]. Furthermore, RRM2 promoted the recurrence and lethality of PCa [38, 39]. Therefore, the roles of CDKN3 and RRM2 have been established in PCa [27, 28] and were not further investigated in this study.

As a member of the INK4/cyclin-dependent kinase inhibitor 2 family, CDKN2C can interact with CDK4 or CDK6 to inhibit the progression of the cell cycle [40]. CDKN2C is upregulated during the transition from prostatic intraepithelial neoplasia to PCa, which may be associated with cell adhesion and invasion of PCa cells [41]. An increasing body of evidence suggested that RACGAP1, a member of the GAP family, plays a vital role in the induction of the division and proliferation of various cells [42, 43]. Importantly, the oncogenic roles of RACGAP1 have been demonstrated in uterine carcinosarcoma, gastric cancer, and hepatocellular carcinoma [44–46]. However, the roles of CDKN2C and RACGAP1 in PCa progression have not been elucidated.

In this study, we found that CDKN2C and RACGAP1 were overexpressed in PCa tissues compared to adjacent tissues, and PCa patients with Gleason score >7 and pathological stage III exhibited higher expression of CDKN2C and RACGAP1 than patients with Gleason score ≤7 and pathological stage II. Additionally, CDKN2C and RACGAP1 knockdown suppressed the proliferation and colony formation of PC3 and C4-2 cells, consistent with the bioinformatics analysis results. Overall, the four E2F-related genes, CDKN2C, CDKN3, RACGAP1, and RRM2, identified in this study, were found to play crucial roles in the progression of PCa, and the E2F-related gene signature based on the four identified genes exhibited good performance in predicting the RFS of PCa patients.

Several limitations were presented in this study. Although an E2F-related gene signature was established and validated by five public RNA-sequencing cohorts, no external validation by real-world cohorts was conducted, which decreases the robustness of our findings to a certain and restricts the clinical application of the E2F-related gene signature. In addition, although the use of the median value of the risk score as the cutoff value could avoid bias between different PCa cohorts, it might not be the optimal threshold.

Importantly, we corroborated that the E2F-related signaling pathway was activated in PCa patients with BCR, and four E2F-related genes were identified as negatively associated with the RFS of PCa patients. We established an E2F-related gene signature based on these genes that showed good performance in predicting biochemically recurrent PCa. Four previously proposed signatures constructed based on more than ten genes were compared with our E2F-related gene signature. Our four-gene signature demonstrated better discriminative ability, highlighting its potential use in clinical practice.

## Conclusions

The E2F signaling pathway plays an important role in biochemically recurrent PCa, and the established E2F-related gene signature and nomogram performed well in RFS prediction, which may serve as a novel prognostic factor for this patient population.

## Supplementary Information

**Additional file 1: Table S1.** Sequences of siRNAs for transfection.

**Additional file 2: Table S2. **The GSVA results of TCGA-PRAD dataset.

**Additional file 3: Table S3.** Supplemental Table 3. The GSVA results of MSKCC (GSE21032) dataset.

**Additional file 4: Table S4.** The GSVA results of GSE116918 dataset.

**Additional file 5: Table S5.** The GSVA results of GSE70768 dataset.

**Additional file 6: Table S6.** The GSVA results of GSE70769 dataset.

**Additional file 7: Table S7.** The results of univariate Cox regression analysis in the MSKCC cohort.

**Additional file 8: Figure S1.** The expression of CDKN2C and RACGAP1 in PCa tissues. Immunohistochemistry analysis showed that CDKN2C and RACGAP1 were more highly expressed in PCa tissues than in adjacent tissues (A, B).

## Data Availability

The datasets used and/or analyzed during the current study are available from the corresponding author on reasonable request.

## Abbreviations

ATCC: American Type Culture Collection
BCR: Biochemical recurrence
CDKN2C: Cyclin-dependent kinase inhibitor 2C
CDKN3: Cyclin-dependent kinase inhibitor 3
CRPC: Castration-resistant prostate cancer
DEPDC1: DEP domain containing 1
GEO: Gene expression omnibus
GSEA: Gene set enrichment analysis
GSVA: Gene set variation analysis
NC: Negative control
OS: Overall survival
PCa: Prostate cancer
PSA: Prostate-specific antigen
RFS: Recurrence-free survival
ROC: Receiver operating characteristic
RACGAP1: Rac GTPase-activating protein 1
RP: Radical prostatectomy
RRM2: Ribonucleotide reductase small subunit M2
RT: Radiation therapy
SDS: Sodium dodecyl sulfate
siRNAs: Small interfering RNAs
TCGA: The Cancer Genome Atlas
TCGA-PRAD: TCGA-prostate adenocarcinoma

## References

[1] Bray F, Ferlay J, Soerjomataram I, Siegel RL, Torre LA, Jemal A (2018). Global cancer statistics 2018: GLOBOCAN estimates of incidence and mortality worldwide for 36 cancers in 185 countries. CA Cancer J Clin.

[2] Kazama A, Saito T, Takeda K, Kobayashi K, Tanikawa T, Kanemoto A, et al. Achieving PSA < 0.2 ng/ml before Radiation Therapy Is a Strong Predictor of Treatment Success in Patients with High-Risk Locally Advanced Prostate Cancer. Prostate Cancer. 2019;2019:4050352. 10.1155/2019/4050352 PMC6854218 31772776

[3] Ying J, Wang CJ, Yan J, Liauw SL, Straka C, Pistenmaa D (2017). Long-term outcome of prostate cancer patients who exhibit biochemical failure despite salvage radiation therapy after radical prostatectomy. Am J Clin Oncol.

[4] Uchio EM, Aslan M, Wells CK, Calderone J, Concato J (2010). Impact of biochemical recurrence in prostate cancer among US veterans. Arch Intern Med.

[5] Artibani W, Porcaro AB, De Marco V, Cerruto MA, Siracusano S (2018). Management of biochemical recurrence after primary curative treatment for prostate cancer: a review. Urol Int.

[6] Zhang Y, Glass A, Bennett N, Oyama KA, Gehan E, Gelmann EP (2004). Long-term outcomes after radical prostatectomy performed in a community-based health maintenance organization. Cancer.

[7] Chen HZ, Tsai SY, Leone G (2009). Emerging roles of E2Fs in cancer: an exit from cell cycle control. Nat Rev Cancer.

[8] Swiss VA, Casaccia P (2010). Cell-context specific role of the E2F/Rb pathway in development and disease. Glia.

[9] Tsantoulis PK, Gorgoulis VG (2005). Involvement of E2F transcription factor family in cancer. Eur J Cancer.

[10] Wu Z, Zheng S, Yu Q (2009). The E2F family and the role of E2F1 in apoptosis. Int J Biochem Cell Biol.

[11] Jiang H, Martin V, Gomez-Manzano C, Johnson DG, Alonso M, White E (2010). The RB-E2F1 pathway regulates autophagy. Cancer Res.

[12] De Meyer T, Bijsmans IT, Van de Vijver KK, Bekaert S, Oosting J, Van Criekinge W (2009). E2Fs mediate a fundamental cell-cycle deregulation in high-grade serous ovarian carcinomas. J Pathol.

[13] Huang CL, Liu D, Nakano J, Yokomise H, Ueno M, Kadota K (2007). E2F1 overexpression correlates with thymidylate synthase and survivin gene expressions and tumor proliferation in non small-cell lung cancer. Clin Cancer Res.

[14] Huang L, Chen K, Cai ZP, Chen FC, Shen HY, Zhao WH (2017). DEPDC1 promotes cell proliferation and tumor growth via activation of E2F signaling in prostate cancer. Biochem Biophys Res Commun.

[15] Choi W, Ochoa A, McConkey DJ, Aine M, Höglund M, Kim WY (2017). Genetic alterations in the molecular subtypes of bladder cancer: illustration in the cancer genome atlas dataset. Eur Urol.

[16] Meng J, Zhou Y, Lu X, Bian Z, Chen Y, Zhou J (2021). Immune response drives outcomes in prostate cancer: implications for immunotherapy. Mol Oncol.

[17] Hänzelmann S, Castelo R, Guinney J (2013). GSVA: gene set variation analysis for microarray and RNA-seq data. BMC Bioinformatics.

[18] Subramanian A, Tamayo P, Mootha VK, Mukherjee S, Ebert BL, Gillette MA (2005). Gene set enrichment analysis: a knowledge-based approach for interpreting genome-wide expression profiles. Proc Natl Acad Sci U S A.

[19] Friedman J, Hastie T, Tibshirani R (2010). Regularization paths for generalized linear models via coordinate descent. J Stat Softw.

[20] Heagerty PJ, Zheng Y (2005). Survival model predictive accuracy and ROC curves. Biometrics.

[21] Yang L, Roberts D, Takhar M, Erho N, Bibby BAS, Thiruthaneeswaran N (2018). Development and validation of a 28-gene hypoxia-related prognostic signature for localized prostate cancer. EBioMedicine.

[22] Liu B, Li X, Li J, Jin H, Jia H, Ge X (2020). Construction and validation of a robust cancer stem cell-associated gene set-based signature to predict early biochemical recurrence in prostate cancer. Dis Markers.

[23] Cuzick J, Swanson GP, Fisher G, Brothman AR, Berney DM, Reid JE (2011). Prognostic value of an RNA expression signature derived from cell cycle proliferation genes in patients with prostate cancer: a retrospective study. Lancet Oncol.

[24] Zhang C, Chen T, Li Z, Liu A, Xu Y, Gao Y, et al. Depiction of tumor stemlike features and underlying relationships with hazard immune infiltrations based on large prostate cancer cohorts. Brief Bioinform. 2021;22(3). 10.1093/bib/bbaa211 32856039

[25] Yin Y, Xu L, Chang Y, Zeng T, Chen X, Wang A (2019). N-Myc promotes therapeutic resistance development of neuroendocrine prostate cancer by differentially regulating miR-421/ATM pathway. Mol Cancer.

[26] Zhang M, Sun Y, Meng J, Zhang L, Liang C, Chang C (2019). Targeting AR-Beclin 1 complex-modulated growth factor signaling increases the antiandrogen Enzalutamide sensitivity to better suppress the castration-resistant prostate cancer growth. Cancer Lett.

[27] Yu C, Cao H, He X, Sun P, Feng Y, Chen L (2017). Cyclin-dependent kinase inhibitor 3 (CDKN3) plays a critical role in prostate cancer via regulating cell cycle and DNA replication signaling. Biomed Pharmacother.

[28] Mazzu YZ, Armenia J, Chakraborty G, Yoshikawa Y, Coggins SA, Nandakumar S (2019). A novel mechanism driving poor-prognosis prostate cancer: overexpression of the DNA repair gene, ribonucleotide reductase small subunit M2 (RRM2). Clin Cancer Res.

[29] Wei L, Wang J, Lampert E, Schlanger S, DePriest AD, Hu Q (2017). Intratumoral and intertumoral genomic heterogeneity of multifocal localized prostate cancer impacts molecular classifications and genomic prognosticators. Eur Urol.

[30] Byron SA, Van Keuren-Jensen KR, Engelthaler DM, Carpten JD, Craig DW (2016). Translating RNA sequencing into clinical diagnostics: opportunities and challenges. Nat Rev Genet.

[31] Johnson DG, Degregori J (2006). Putting the oncogenic and tumor suppressive activities of E2F into context. Curr Mol Med.

[32] Ramos-Montoya A, Lamb AD, Russell R, Carroll T, Jurmeister S, Galeano-Dalmau N (2014). HES6 drives a critical AR transcriptional programme to induce castration-resistant prostate cancer through activation of an E2F1-mediated cell cycle network. EMBO Mol Med.

[33] Zhou Q, Wang C, Zhu Y, Wu Q, Jiang Y, Huang Y (2019). Key genes and pathways controlled By E2F1 in human castration-resistant prostate cancer cells. Onco Targets Ther.

[34] Zhao J, Wu XY, Ling XH, Lin ZY, Fu X, Deng YH (2013). Analysis of genetic aberrations on chromosomal region 8q21-24 identifies E2F5 as an oncogene with copy number gain in prostate cancer. Med Oncol.

[35] Chang SL, Chen TJ, Lee YE, Lee SW, Lin LC, He HL (2018). CDKN3 expression is an independent prognostic factor and associated with advanced tumor stage in nasopharyngeal carcinoma. Int J Med Sci.

[36] Kretschmer C, Sterner-Kock A, Siedentopf F, Schoenegg W, Schlag PM, Kemmner W (2011). Identification of early molecular markers for breast cancer. Mol Cancer.

[37] Xu X, Page JL, Surtees JA, Liu H, Lagedrost S, Lu Y (2008). Broad overexpression of ribonucleotide reductase genes in mice specifically induces lung neoplasms. Cancer Res.

[38] Mazzu YZ, Armenia J, Nandakumar S, Chakraborty G, Yoshikawa Y, Jehane LE (2020). Ribonucleotide reductase small subunit M2 is a master driver of aggressive prostate cancer. Mol Oncol.

[39] Huang Y, Liu X, Wang YH, Yeh SD, Chen CL, Nelson RA (2014). The prognostic value of ribonucleotide reductase small subunit M2 in predicting recurrence for prostate cancers. Urol Oncol.

[40] Zhu L, Lu Z, Zhao H (2015). Antitumor mechanisms when pRb and p53 are genetically inactivated. Oncogene.

[41] Ashida S, Nakagawa H, Katagiri T, Furihata M, Iiizumi M, Anazawa Y (2004). Molecular features of the transition from prostatic intraepithelial neoplasia (PIN) to prostate cancer: genome-wide gene-expression profiles of prostate cancers and PINs. Cancer Res.

[42] Zhao WM, Fang G (2005). MgcRacGAP controls the assembly of the contractile ring and the initiation of cytokinesis. Proc Natl Acad Sci U S A.

[43] Hirose K, Kawashima T, Iwamoto I, Nosaka T, Kitamura T (2001). MgcRacGAP is involved in cytokinesis through associating with mitotic spindle and midbody. J Biol Chem.

[44] Mi S, Lin M, Brouwer-Visser J, Heim J, Smotkin D, Hebert T (2016). RNA-seq identification of RACGAP1 as a metastatic driver in uterine carcinosarcoma. Clin Cancer Res.

[45] Saigusa S, Tanaka K, Mohri Y, Ohi M, Shimura T, Kitajima T (2015). Clinical significance of RacGAP1 expression at the invasive front of gastric cancer. Gastric Cancer.

[46] Wang SM, Ooi LL, Hui KM (2011). Upregulation of Rac GTPase-activating protein 1 is significantly associated with the early recurrence of human hepatocellular carcinoma. Clin Cancer Res.

