# Supplementary material for: Identification and validation of an E2F-related gene signature for predicting recurrence-free survival in human prostate cancer
Source: Cancer Cell Int. 2022 Dec 5;22:382. doi: 10.1186/s12935-022-02791-9 (PMC9721026; doi:10.1186/s12935-022-02791-9)
Supplement: Supplementary file 1 — Additional file 1: Table S1. Sequences of siRNAs for transfection. [file 12935_2022_2791_MOESM1_ESM.docx]

**Supplemental Table 1. Sequences of siRNAs for transfection.**

| Genes |  | Sequences |
| --- | --- | --- |
| si- CDKN2C | F | CTATGGGAGGAATGAGGTTGT |
|  | R | ACAACCTCATTCCTCCCATAG |
| si- RACGAP1 | F | CAGGTGGATGTAGAGATCAAA |
|  | R | TTTGATCTCTACATCCACCTG |
| Nontargeting control (NC) | F | UUCUCCGAACGUGUCACGUTT |
|  | R | ACGUGACACGUUCGGAGAATT |
