# Supplementary material for: Identification and validation of an E2F-related gene signature for predicting recurrence-free survival in human prostate cancer
Source: Cancer Cell Int. 2022 Dec 5;22:382. doi: 10.1186/s12935-022-02791-9 (PMC9721026; doi:10.1186/s12935-022-02791-9)
Supplement: Supplementary file 8 — Additional file 8: Figure S1. The expression of CDKN2C and RACGAP1 in PCa tissues. Immunohistochemistry analysis showed that CDKN2C and RACGAP1 were more highly expressed in PCa tissues than in adjacent tissues (A, B). [file 12935_2022_2791_MOESM8_ESM.pdf]

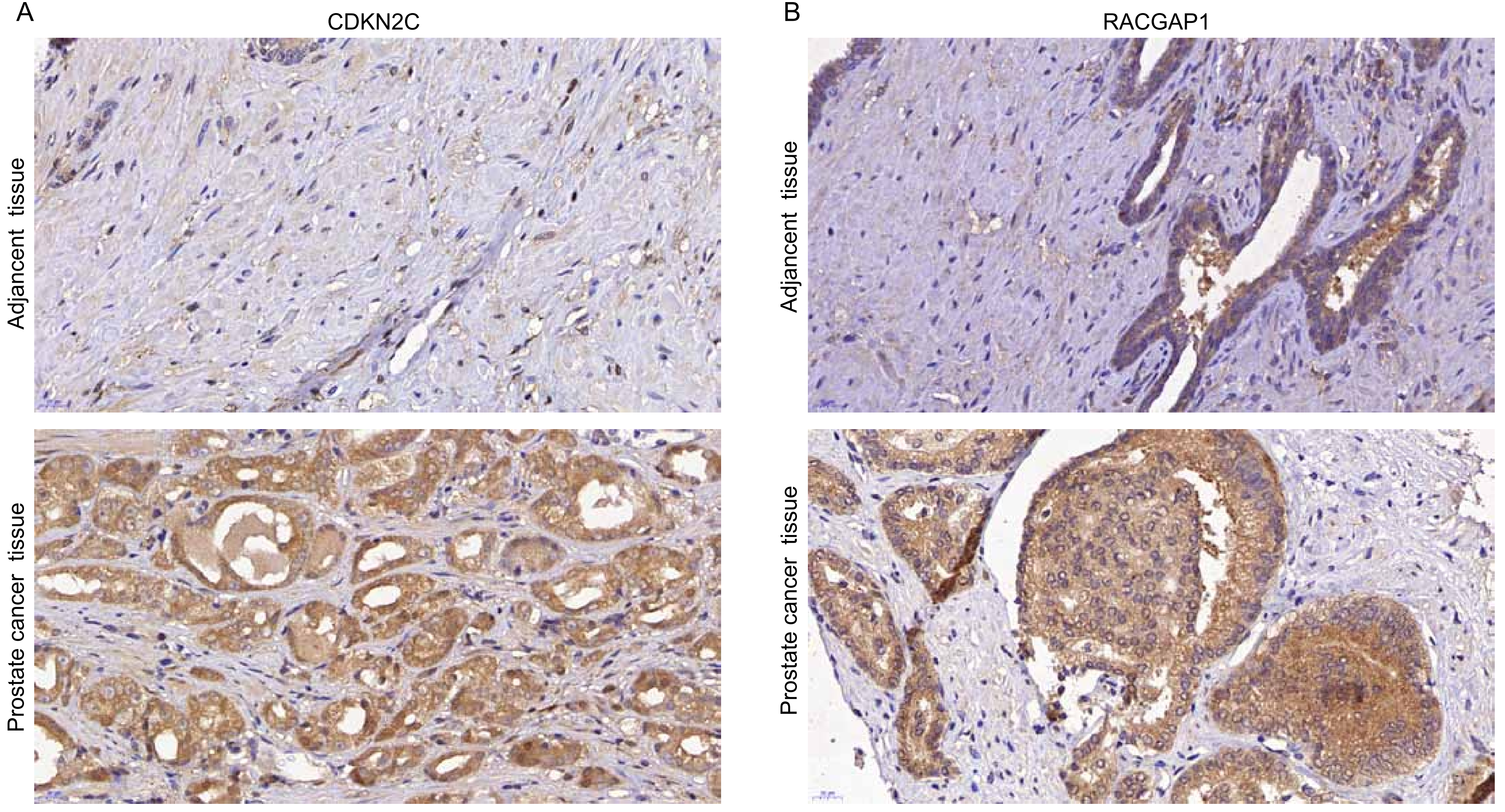
A
CDKN2C
B
RACGAP1
Adjancent tissue
Prostate cancer tissue
Adjancent tissue
Prostate cancer tissue
